# Mesenchymal Stem Cells Alleviate Renal Fibrosis and Inhibit Autophagy via Exosome Transfer of miRNA-122a

**DOI:** 10.1155/2022/1981798

**Published:** 2022-07-07

**Authors:** Dawei Li, Junwen Qu, Xiaodong Yuan, Shaoyong Zhuang, Haoyu Wu, Ruoyang Chen, Jiajin Wu, Ming Zhang, Liang Ying

**Affiliations:** Department of Urology, Renji Hospital, School of Medicine, Shanghai Jiao Tong University, Shanghai, China

## Abstract

Exosomes derived from mesenchymal stem cell (MSC) alleviate kidney damage through autophagy. This study determined whether MSCs relieve renal fibrosis and inhibit autophagy by exosome transfer of miRNA-122a. The gene expression involved in the mTOR signaling pathway and autophagy was assessed in TGF-*β*1-treated human renal tubular epithelial cells (HK-2) and unilateral ureteral obstruction (UUO) mice before and after MSC-derived exosomes and miRNA-122a mimic treatment. Small RNA (sRNA) next-generation sequencing was also performed on TGF-*β*1-treated HK-2 cells. MSC-derived exosomes relieve fibrosis caused by TGF*β* in HK-2 via regulation of the mTOR signaling pathway and downstream autophagy. Furthermore, we found that MSC-derived exosomes mediate miRNA-122a to relieve renal fibrosis in HK-2 cells in response to TGF-*β*1 through the regulation of mTOR signaling and autophagy. In the UUO mouse model, miRNA-122a mimic-transfected MSC treatment and its combination with 3-MA both recapitulated the same results as the in vitro experiments, along with reduced expansion of renal tubule, interstitial expansion, and preservation of kidney architecture. The antifibrotic activity of MSC-derived exosomes after renal fibrosis occurs partially by autophagy suppression via excreted exosomes containing mainly miRNA-122a. These findings indicate that the export of miRNA-122a via MSC-derived exosomes represents a novel strategy to alleviate renal fibrosis.

## 1. Introduction

The incidence and fatality rate of chronic kidney disease is increasing globally, and it has become a public health problem affecting billions of people worldwide [1]. In recent years, the efficient application of autologous mesenchymal stem cells (MSCs) has provided unprecedented effectiveness for the treatment of chronic kidney disease or delayed the progression of chronic kidney disease [2, 3]. MSCs have been shown to reduce acute and chronic kidney injury in several animal models and a number of clinical trials related to kidney disease [4–6].

It has been shown that the major target of MSC treatment is the paracrine mechanism, rather than the MSC itself [7, 8]. Evidence shows that MSC-derived exosomes can interact with recipient cells and transport their contents to the cytosol of recipient cells, such as functional proteins, translatable mRNA, and their miRNA [9]. Studies have shown that exosomes not only are active participants in suppressing renal fibrosis but also represent a potential therapeutic carrier for the treatment of chronic kidney disease by ameliorating renal fibrosis [10, 11]. Gallo et al. demonstrated that MSC-derived exosomes can interfere with the mesangial cell collagen production in a hyperglycemic environment [12]. An in vivo study indicated that injecting exosomes secreted by MSCs overexpressing miRNA-let7c through the tail vein of unilateral ureteral obstruction (UUO) mice alleviated kidney damage [13]. Another study showed that MSC-derived exosomes reversed the deterioration of renal function in a UUO model by improving UUO renal endothelial cell-to-interstitial transformation and improving the sparse capillaries around renal tubules [14]. Therefore, these experiments suggest that the antifibrotic effect of exosomes is mediated, at least in part, by the transfer of miRNAs that regulate targets related to renal fibrosis.

Autophagy is a metabolic pathway that degrades long-lived proteins and damaged organelles through lysosomes, and plays an important role in the response to cellular stress [15]. Autophagy plays an important role in the treatment of diseases with MSCs from different sources. It has been shown that MSC-derived exosomes alleviate kidney damage in rats through autophagy mediated by the mTORC1 pathway [16]. Recent studies have shown that autophagy may be a potential regulatory mechanism in the occurrence and development of renal interstitial fibrosis [17, 18]. However, the results of studies on the role of autophagy in renal interstitial fibrosis are not consistent. Furthermore, the mechanism through which miRNAs secreted by MSC-derived exosomes affect renal fibrosis and the role of autophagy in this process remain unclear. Clinical and experimental studies clearly show that miRNA-122 is a marker of fibrogenesis, cardiovascular damage, and dysfunction. However, there are few reports regarding its role in renal fibrosis [19]. Therefore, we performed in vivo and in vitro experiments to determine whether the relief of renal fibrosis by MSC-derived exosome treatment is mediated by miRNA-122a. We also determined the role of mTOR signaling and changes in the autophagic response in renal fibrosis.

## 2. Methods

### 2.1. MSC Culture and Identification

Bone mesenchymal stem cells (MSCs) were obtained from Cyagen Biosciences (Guangzhou, China) and maintained in Dulbecco's modified Eagle medium (DMEM, Gibco) supplemented with 10% fetal bovine serum (FBS) and 1% streptomycin sulfate and penicillin (Gibco) in a humidified atmosphere of 95% air and 5% CO_2_ at 37°C until confluent. The cell suspensions (1 × 10^5^ cells in 100 *μ*l) of MSCs were incubated with fluorescent-labeled specific antibodies (CD34, CD45, CD44, and CD90, Abcam) that bind to cells expressing a specific cell surface marker. IgG antibody (Abcam) was used as a control for nonspecific staining. After incubation at room temperature in the dark for 30 min, the cells were washed three times with phosphate-buffered saline (PBS). The surface antigen expression of MSCs was detected by flow cytometry (FC500, Beckman Coulter, USA) (Figure S1A).

### 2.2. MSC Exosome Preparation, Identification, Uptake, and Inhibition

MSCs were maintained in MSC media as described above. After growing to 80% confluence, MSC cells were washed with PBS and then cultured in serum-free DMEM for 24 h, followed by the isolation of MSC exosomes from the supernatant of the cultures with ExoQuick exosome precipitation solution (System Bioscience, Mountain View, CA). The exosomes were quantified using the BCA assay, size was measured by nanoparticle tracking analysis, and the morphological characteristics were determined by transmission electron microscopy (TEM) (Figure S1B–E).

To demonstrate that MSC-derived exosomes were taken up by cultured HK-2 cells, the latter were incubated with PKH26-labeled exosomes for 24 h and observed under a confocal fluorescent microscope. The method of labeling exosomes was performed according to a previous study [13].

For exosomal inhibition, MSC cells were cultured in an MSC medium supplemented with 20 *μ*M GW4869 (Sigma) (MSC supernatant+GW4869) for 24 h and then replaced with serum-free MSC medium containing 20 *μ*M GW4869 for 24 h prior to exosome isolation.

### 2.3. HK-2 Treatment with MSC Exosomes

MSCs at a density of 1 × 10^5^ cells/well were seeded and cultured with MSC media as previously described at 37°C and of 95% air and 5% CO_2_ for 24 h. MSC exosomes were isolated from the supernatants of the cultures using the ExoQuick exosome precipitation solution (System Bioscience, Mountain View, CA). Human renal tubular epithelial cells (HK-2, FuHeng) were cultured in DMEM (Gibco) supplemented with 10% FBS, 4 mM L-glutamine, and 1% streptomycin sulfate and penicillin and cultured with or without TGF-*β*1 (5 ng/ml; R&D Systems) in a humidified atmosphere of 95% air and 5% CO_2_ at 37°C at 37°C and 5% CO_2_. The HK-2 cells were plated into 6-well Transwell chambers (4 *μ*m, Corning) at a density of 1 × 10^5^ cells/well, followed by the addition of 2 ml supernatants containing MSC-derived exosomes (2 *μ*g/ml) for 24 h.

### 2.4. RNA Quantification and sRNA Library Preparation and Sequencing

The total RNA was extracted from TGF-*β*1-treated HK-2 cells incubated with MSC+GW4869 (GW4869-1, GW4869-2, and GW4869-3) and treated with MSC-exo (exo-1, exo-2, and exo-3). RNA degradation and contamination, especially DNA contamination, were monitored on 1.5% agarose gels. RNA concentration and purity were measured using a NanoDrop 2000 Spectrophotometer (Thermo Fisher Scientific, Wilmington, DE). RNA integrity was assessed using the RNA Nano 6000 Assay Kit and the Agilent Bioanalyzer 2100 System (Agilent Technologies, CA, USA). A total of 2.5 ng RNA per sample was used as the input material for RNA sample preparation. Sequencing libraries were prepared using NEBNext® Ultra™ small RNA Sample Library Prep Kit for Illumina® (NEB, USA) following the manufacturer's instructions, and index codes were added to attribute sequences to each sample. The clustering of the index-coded samples was performed on a cBot Cluster Generation System using TruSeq PE Cluster Kitv3-cBot-HS (Illumina) according to the manufacturer's instructions. After the cluster generation, sequencing was performed with an Illumina SE50 system at Biomarker Technologies Co., Ltd. (Beijing, China).

### 2.5. Bioinformatics Analysis of Sequencing Data

Raw reads of the fastq format were initially processed through in-house Perl scripts, and clean reads were obtained by removing the low-quality reads. Reads lacking adapter and the ones with ploy-N and lengths smaller than 15 nt or longer than 35 nt were also removed. At the same time, Q20, Q30, and GC content of the cleaned data were calculated. All the downstream analyses were based on high-quality clean data. The clean reads were subjected to the Silva database, GtRNAdb database, Rfam and ATG7 siRNA database, and Repbase database sequence alignment to filter ribosomal RNA (rRNA), transfer RNA (tRNA), small nuclear RNA (snRNA), small nucleolar RNA (snoRNA), and other ncRNA and repeats.

After the unannotated reads containing miRNA were generated, they were blasted against miRBase to search for known miRNAs. Potential novel miRNAs were predicted using the miRdeep2 program. Randfold tools soft was used to predict novel miRNA secondary structure. The miFam.dat (http://www.miRbase.org/ftp.shtml) was used to determine the miRNA family of the miRNAs. miRNA expression was calculated and normalized by transcript per million. The read counts were adjusted by the edgeR program package using a one-scaling normalized factor. Differential expression analysis was performed using the EBSeq (2010) R package. False discovery rate < 0.05 and |Log2 (fold change) | ≥ 1 was set as the threshold for significantly differential expression.

### 2.6. Preparation of miRNA-122a Mimic/Inhibitor

The miRNA-122a mimic/inhibitor and miRNA-NC (negative control) were obtained from RiboBio and diluted with 0.9% NaCl to obtain a 25 *μ*M stock solution.

### 2.7. HK-2 Cell Transfection

TGF-*β*1-treated HK-2 cells were transfected with 25 nM miRNA-122a mimic or 25 nM miRNA-NC with or without ATG7 siRNA (50 nM concentration of siRNA). Successful transfection was assessed by qPCR measurement of ATG7 mRNA levels. After 24 h, the transfected cells were maintained in a medium containing 10% FBS and treated with 5 mM 3-MA for 30 min. The protein and mRNA levels of TGFBR1, *α*-SMA, Col1a1, fibronectin, and E-cadherin were determined by western blot analysis and qPCR. In addition, protein levels of LC3II/I, p62, Beclin 1, and ATG7 were measured by western blot analysis.

### 2.8. Autophagic Flux

TGF-*β*1-treated HK-2 cells were cultured on coverslips for 24 h, transfected with adenoviruses expressing RFP-GFP-LC3 (MOI 20, Asia-Vector biotechnology, China), and then maintained in serum-free DMEM. After 6 hours, the cells were fixed with 4% paraformaldehyde (PFA) and autophagic flux was observed under a confocal fluorescence microscope (FV1200, OLYMPUS). The number of autophagosomes and autolysosomes was quantified in each cell (20–30 cells per group) in three independent experiments.

### 2.9. Animals

Male C57BL/6J mice (8–12 weeks old) weighing 20–25 g was purchased from jh-labanimal (Shanghai) and randomly divided into a sham group (*n* = 5) or groups treated with vehicle, MSCs, miRNA-122a mimic-transfected MSCs, or miRNA-122a mimic-transfected MSCs+3-MA (*n* = 10/group). The mice were anesthetized by injection with 50 mg/kg of Zoletil 50 (Virbac Laboratories, Carros, France) before the UUO surgery. The UUO surgery was conducted as previously described [13]. After surgery, mice in the treatment groups were intravenously administered PBS vehicle, 1 × 10^6^ MSCs, 1 × 10^6^ miRNA-122a mimic-transfected MSCs, or 1 × 10^6^ miRNA-122a mimic-transfected MSCs+3-MA by tail vein injection, followed by intraperitoneal injection with 30 mg/kg 3-MA. The experiments were performed according to the Guide for the Care and Use of Laboratory Animals, and permission was obtained from the Renji Hospital Animal Ethics Committee. The mice received commercial chow and were maintained in a 12 h light/dark cycle.

### 2.10. Detection of TGF-*β*1 and Urea Levels

On the 14th day, all the mice fasted for 4 h before getting administered with Zoletil 50 anesthesia. Blood was then collected from the ophthalmic artery, and serum was obtained by centrifugation at 4000 rpm for 20 min at 4°C. Serum TGF-*β*1 level was measured immediately using an enzyme-linked immunosorbent assay (LiankeBio, China) and colorimetric assay (Nan Jing Jian Chen Biotechnology, China), respectively, according to the manufacturer's instructions.

### 2.11. Histological Analyses and Immunohistochemistry

Kidneys were fixed with 10% neutral-buffered formaldehyde and embedded in paraffin, cut into 3–5 mm-thick slices, and examined by histopathology and immunohistochemistry. To assess changes in kidney structure, hematoxylin and eosin staining was performed. Tissue sections were stained with hematoxylin (4 min), differentiated with acid alcohol, and counterstained with eosin for 2 min. The histopathological scoring for parameters of tubular cell necrosis, infiltration of inflammatory cells, tubular dilation, and cast formation was performed by two different pathologists. Images were obtained using a fluorescence microscope (Nikon, Japan).

For immunohistochemistry, tissue sections were deparaffinized and dehydrated, followed by incubation in 3% hydrogen peroxide for 5 min and blocking solution (5% bovine serum albumin and PBS containing 0.5% Tween 20, pH 7.5) for 30 min. The sections were then incubated with primary antibodies against fibronectin (1 : 200; Abcam) and *α*-SMA (1 : 1000; Abcam) at 4°C overnight followed by incubation with HRP-conjugated secondary antibody. The sections were observed under a fluorescence microscope (Nikon, Japan).

### 2.12. Quantitative Real-Time PCR

Total RNA was isolated using a Trizol reagent (Invitrogen), and RNA concentration was measured by spectrophotometry. Total RNA was reverse transcribed using TransScript All-in-One First-Strand cDNA Synthesis kit (TransGen Biotech, China). The PCR reaction was performed with SG Fast qPCR Master Mix (Sangon, China) using an ABI StepOnePlus Real-Time PCR System. The program included 3 min at 95°C followed by 45 melting (7 s at 95°C), annealing (10 s at 57°C), and extending (15 s at 72°C) cycles. The relative gene expression of the target genes was normalized to GAPDH expression and calculated by the 2–^*ΔΔ*CT^ method and relative to control samples, which were assigned a value of 1. Each measurement was conducted using three replicates per group. The primers for the target genes are listed in Table S1.

### 2.13. Protein Preparation and Western Blot Analysis

Total protein was extracted using RIPA Lysis Buffer (Beyotime, Haimen, China). The protein concentration of the lysates was quantified with a BCA protein assay kit (Beyotime, Haimen, China). Denatured proteins (40 *μ*g) were loaded onto SDS-PAGE gels, and the separated proteins were transferred to PVDF membranes followed by blocking in 5% nonfat milk for 30 min. The PVDF membranes were incubated with primary antibody (Table S2) at 4°C overnight and washed with TBST six times for 5 min each. Membranes were then incubated with anti-rabbit Ig-HRP-conjugated antibody (1 : 5000 dilution) at 37°C for 2 h. The bands were visualized by enhanced chemiluminescence (ECL system; Amersham, Arlington Heights, IL) and quantified with ImageJ v1.51 (National Institutes of Health, USA) software. The density of the target bands was normalized to GAPDH before comparing the groups.

### 2.14. Transmission Electron Microscopy

To observe ultrastructure alterations, TEM analysis was performed on tissue sections. For images of kidney tissue, samples were fixed with electron microscope fixative (Servicebio, China) for 2–4 h, washed for 15 min three times in 0.1 M PBS, postfixed with 1% OsO4 for 1 h, washed for 15 min three times in 0.1 M PBS, and dehydrated in a graded ethanol series (50%, 70%, 80%, 90%, 95%, and 100%, 15 min each) and in anhydrous ethanol and acetone at room temperature (two times, 15 min each). Samples were soaked in acetone and Epon 812 resin (1 : 1) for 2–4 h, followed by acetone and Epon 812 resin (2 : 1) overnight, and the samples were soaked in pure Epon 812 resin for 5 h. They were transferred to an embedding tube overnight at 37°C and allowed to polymerize for 48 h at 60°C. Each sample was cut into approximately 60–80 nm slices with a Leica UC7 ultrathin slice machine and dyed using uranium acetate and lead citrate. Images of the slices were randomly captured using TEM with a field-emission gun operating at 200 kV (Tecnai G2F20, FEI Company, USA).

### 2.15. Statistical Analysis

The data are presented as the mean ± SEM (Standard Error of the Mean) from at least three independent experiments. Repeated measures ANOVA with Sidak's post hoc test was used to assess statistical significance by using the Spearman rank method and Statistical SPSS version 19.0 software (IBM, Chicago, USA). *P* < 0.05 was considered statistically significant. Bar plots were created by using GraphPad Prism 8.0.

## 3. Results

### 3.1. MSC-Derived Exosomes Relieve Fibrosis Caused by TGF*β* in HK-2 via Regulation of the mTOR Signaling Pathway and Downstream Autophagy

**Figure 1:**
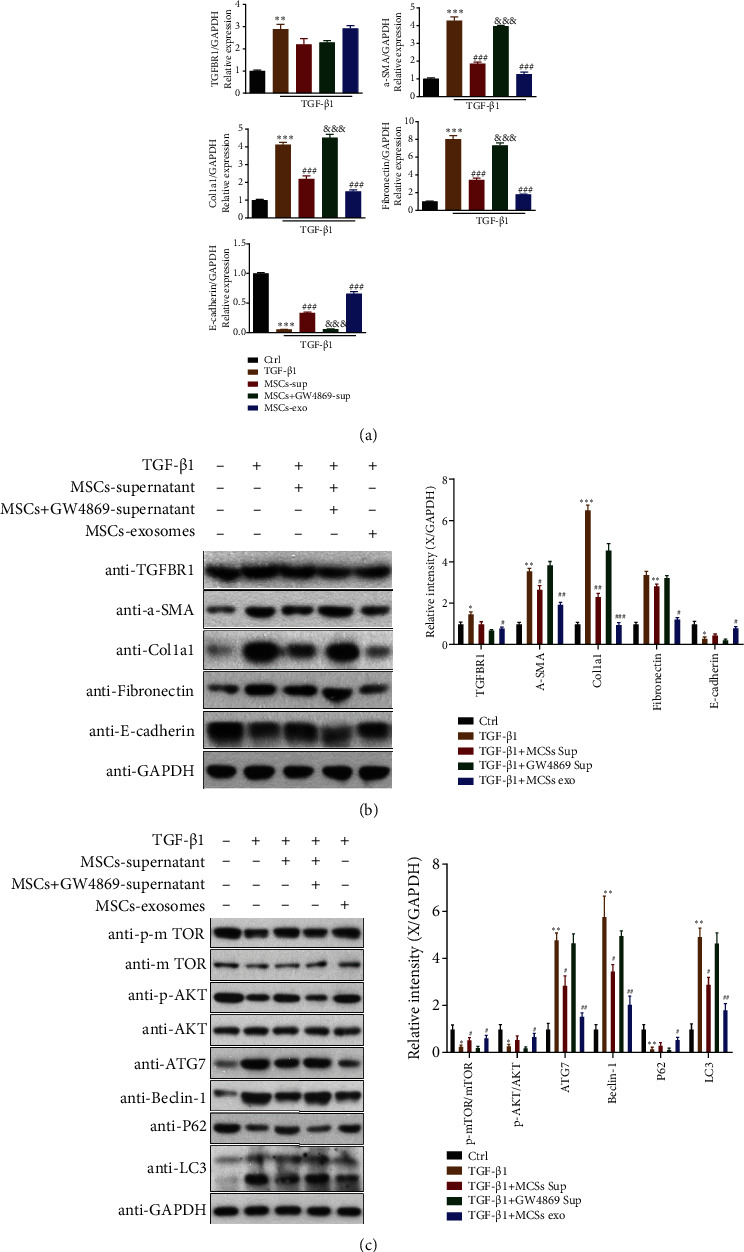
MSC-derived exosomes relieve fibrosis caused by TGF*β* in HK-2 cells via regulation of mTOR signaling and downstream autophagy. (a) TGFBR1, *α*-SMA, Col1a1, fibronectin, and E-cadherin mRNA levels were evaluated by qPCR in HK-2 cells. (b) Immunoblotting analysis for TGFBR1, *α*-SMA, Col1a1, fibronectin, and E-cadherin expression in HK-2 cells. Quantitative analysis of TGFBR1, *α*-SMA, Col1a1, fibronectin, and E-cadherin expression is shown on the right (*n* = 3 independent experiments). (c) LC3 II/I, p62, Beclin 1, ATG7, mTOR, p-mTOR, AKT, and p-AKT protein expression was evaluated by western blotting in HK-2 cells, and the quantitative analysis is shown on the right (*n* = 3 independent experiments). Results are expressed as the mean ± SEM. ^∗∗∗^*P* < 0.001, ^∗∗^*P* < 0.01, and ^∗^*P* < 0.05.

The TGF-*β* signaling pathway is a vital mediator in the development of renal fibrosis. To study the effect of MSC-derived exosomes on renal fibrosis, HK-2 cells were treated with TGF-*β*1 (5 ng/ml) for 48 h. The expression of TGFBR1 and related fibrotic genes (*α*-SMA, Col1a1, fibronectin, and E-cadherin) was determined by qPCR and western blot analysis. Compared with untreated HK-2 cells, TGF-*β*1 treatment markedly upregulated the mRNA and protein levels of TGFBR1, *α*-SMA, Col1a1, and fibronectin in HK-2 cells, whereas E-cadherin expression was downregulated (Figures 1(a) and 1(b)). When TGF-*β*1-treated HK-2 cells were treated with MSC supernatant or MSC-exo, the expression of *α*-SMA, Col1a1, and fibronectin was significantly decreased and E-cadherin was increased. This was more evident in the MSC-exo-treated group (Figures 1(a) and 1(b)). However, GW4869 treatment blocked the expression changes in these genes caused by MSC supernatant in TGF-*β*1-treated HK-2 cells, suggesting that the MSC supernatant exerts its effect on TGF-*β*1-treated HK-2 cells via exosomes. These data indicate that MSC-derived exosomes relieve fibrosis caused by TGF-*β* in kidney tubular epithelial cells.

To investigate whether the reduction of fibrosis in TGF-*β*1-treated HK-2 cells caused by MSC-derived exosome treatment was related to autophagy, the autophagy-related protein levels of LC3II/I, p62, Beclin 1, and ATG7, were assessed. As shown in Figure 1(c), the levels of LC3II/I, Beclin 1, and ATG7 in HK-2 cells were significantly increased, whereas p62 levels were decreased after TGF-*β*1 treatment. These expression levels were attenuated by MSC supernatant or MSC-exo treatment. GW4869 treatment blocked changes in autophagy-related protein levels caused by treatment with MSC supernatant in TGF-*β*1-treated HK-2 cells. These data indicate that MSC-derived exosomes relieve fibrosis caused by TGF-*β* in kidney tubular epithelial cells via autophagy modulation.

To understand the mechanism through which MSC-derived exosomes regulate autophagy, the mTOR signaling pathway was examined. The results showed that MSC-induced autophagic modulation is mediated by the mTOR signaling pathway, which was demonstrated by the upregulated phosphorylation of mTOR (p-mTOR) and AKT (p-AKT) in TGF-*β*1-treatd HK-2 cells (Figure 1(c)).

### 3.2. Identification of Differentially Expressed miRNA Profiles between TGF-*β*1-Treated HK-2 Cells Treated with MSC Supernatant+GW4869 and MSC-Exo

**Figure 2:**
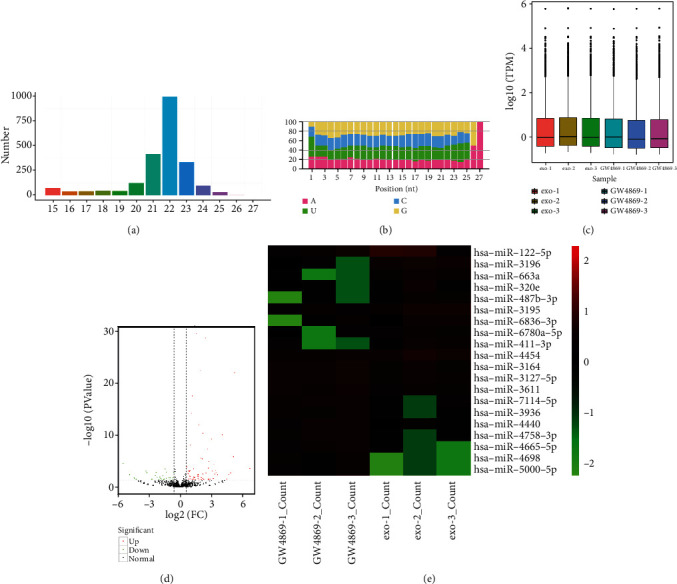
Differentially expressed miRNA profiles between TGF-*β*1-treated HK-2 cells treated with mesenchymal stem cell (MSC) supernatant+GW4869 and MSC-exo. (a) The length distribution of identified miRNAs. (b) The miRNA nucleotide bias at each position. (c) The overall distribution of the expression of each sample. (d) Volcano plot of the identified miRNAs. (e) Hierarchical clustering analysis of differentially expressed miRNAs.

The antifibrotic effects of exosomes are regulated, in part, by the transfer of miRNAs that attenuate targets related to renal fibrosis [14]. To investigate how miRNAs secreted by MSC-derived exosomes affect renal fibrosis, we constructed and sequenced six sRNA libraries from TGF-*β*1-treated HK-2 cells incubated with MSC+GW4869 and MSC-exo. As a result, a total of 2247 miRNAs, including 1570 known miRNAs and 677 novel miRNAs, were identified from six sRNA libraries (Table S3). The distribution of lengths indicated that most identified miRNA reads were 21–24 nt (Figure 2(a), Table S3). The miRNA nucleotide bias at each position is displayed in Figure 2(b). The overall distribution of the expression of each sample is shown in Figure 2(c) (Table S4). A total of 97 miRNAs were identified as differentially expressed between the MSC supernatant+GW4869 and MSC-exo groups, of which 67 miRNAs were upregulated and 30 miRNAs were downregulated after the MSC-exo treatment (Figure 2(d), Table S5). Hierarchical clustering analysis indicated that these differentially expressed miRNA expression profiles were distinguishable between the two groups (Figure 2(e)). Compared with the MSC supernatant+GW4869 group, the top ten upregulated miRNAs in the MSC-exo group are listed in Table 1. hsa-miRNA-122-5p, which belongs to the miR-122 family, was the most markedly upregulated miRNA. Therefore, miRNA-122 was selected for further studies.

### 3.3. MSC-Derived Exosomes Mediate miRNA-122a to Relieve Renal Fibrosis in HK-2 Cells in Response to TGF-*β*1

**Figure 3:**
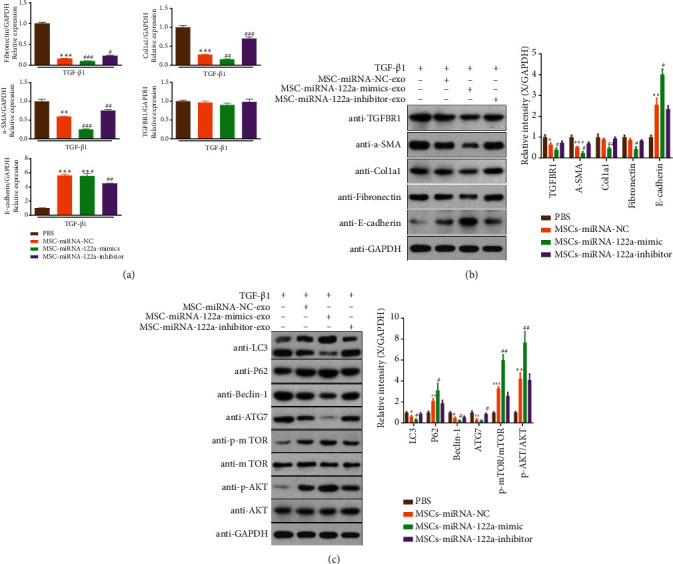
MSC-derived exosomes mediate miRNA-122a to relieve renal fibrosis in HK-2 cells in response to TGF-*β*1. (a) TGFBR1, *α*-SMA, Col1a1, fibronectin, and E-cadherin mRNA levels were measured by qPCR in HK-2 cells. (b) Western blot analysis of TGFBR1, *α*-SMA, Col1a1, fibronectin, and E-cadherin protein expression in HK-2 cells. Quantitative analysis of TGFBR1, *α*-SMA, Col1a1, fibronectin, and E-cadherin protein expression is shown on the right (*n* = 3 independent experiments). (c) LC3 II/I, p62, Beclin 1, ATG7, mTOR, p-mTOR, AKT, and p-AKT protein expression was evaluated by western blotting in HK-2 cells, and their quantitative analysis is shown on the right (*n* = 3 independent experiments). The results are expressed as the mean ± SEM. Compared with the TGF*β* group, ^∗∗∗^*P* < 0.001, ^∗∗^*P* < 0.01, and ^∗^*P* < 0.05. Compared with TGF*β*+MSC- (miRNA-NC) exo, ^###^*P* < 0.001, ^##^*P* < 0.01, and ^#^*P* < 0.05.

miRNA-122a is a key marker of fibrogenesis, cardiovascular damage, and dysfunction [19]. To explore whether differentially expressed miRNA-122a is related to autophagy and derived from exosomes secreted by MSCs, TGF-*β*1-treated HK-2 cells were treated with exosomes derived from MSCs transfected with the miRNA-122a mimic/inhibitor and miR-NC. TGF-*β*1-treated HK-2 cells treated with miR-NC-MSCs resulted in a significant reduction in the expression of *α*-SMA, Col1a1, and fibronectin mRNA and protein expression, whereas E-cadherin expression was enhanced (Figures 3(a) and 3(b)). This suggests that differentially expressed miRNAs related to autophagy are derived from exosomes secreted by MSCs. The changes to these downregulated genes were more evident after treatment with miRNA-122a mimic-transfected MSC-derived exosomes; however, these effects were decreased after cocultivation with exosomes derived from MSCs transfected with the miRNA-122a inhibitor (Figures 3(a) and 3(b)). This indicates that miRNA-122a significantly attenuates the expression of key genes involved in kidney fibrosis in HK-2 cells in response to TGF-*β*1. Additionally, we found that the protein levels of LC3II/I, Beclin 1, and ATG7 in TGF-*β*1-treated HK-2 cells were markedly downregulated and p62, phospho-mTOR, and AKT were upregulated by miRNA-NC-MSCs and miRNA-122a mimic-MSC-derived exosome treatment but were more evident in the miRNA-122a mimic-MSC group (Figure 3(c)). However, these alterations were reversed by exosomes derived from MSCs transfected with the miRNA-122a-inhibitor (Figure 3(c)). These data suggest that MSC-derived exosomes modulate miRNA-122a to relieve renal fibrosis in HK-2 cells in response to TGF-*β*1 through regulation of mTOR signaling and downstream autophagy.

### 3.4. miRNA-122a Relieves Fibrosis Caused by TGF*β* in HK-2 through the Autophagy Signaling Pathway and Autophagic Flux

**Figure 4:**
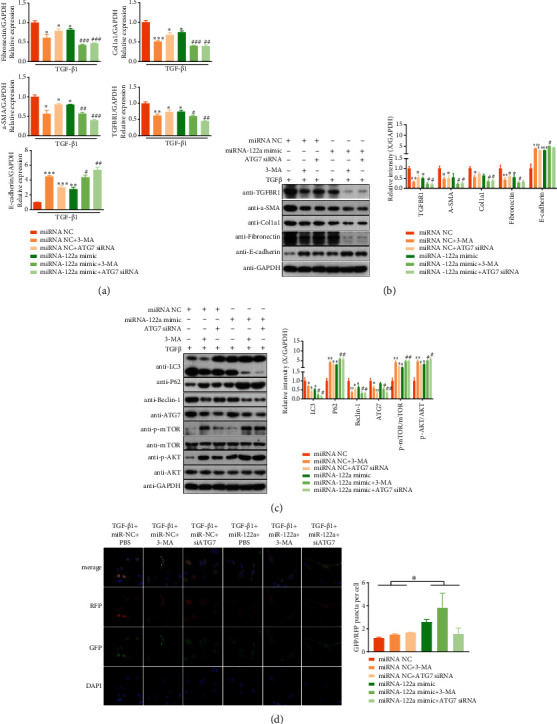
miRNA-122a relieves renal fibrosis caused by TGF*β* in HK-2 cells through autophagy signaling and autophagic flux. (a) TGFBR1, *α*-SMA, Col1a1, fibronectin, and E-cadherin mRNA levels were measured by qPCR in HK-2 cells. (b) Western blotting analysis of TGFBR1, *α*-SMA, Col1a1, fibronectin, and E-cadherin protein expression in HK-2 cells. Quantitative analysis of TGFBR1, *α*-SMA, Col1a1, fibronectin, and E-cadherin protein expression is shown on the right (*n* = 3 independent experiments). (c) LC3II/I, p62, Beclin 1, ATG7, mTOR, p-mTOR, AKT, and p-AKT protein expression was evaluated by western blotting in HK-2 cells, and their quantitative analysis is shown on the right (*n* = 3 independent experiments). (d) The effect of miRNA-122a mimic on autophagy flux after treatment with an autophagy inhibitor in HK-2 cells as detected by confocal scanning laser microscopy. Results are expressed as the mean ± SEM. Compared with the miRNA-NC+TGF*β* group, ^∗∗∗^*P* < 0.001, ^∗∗^*P* < 0.01, and ^∗^*P* < 0.05. Compared with miRNA-122a mimic+TGF*β*, ^###^*P* < 0.001, ^##^*P* < 0.01, and ^#^*P* < 0.05.

To further confirm whether miRNA-122a affects renal fibrosis through the autophagy signaling pathway, we used 3-MA and an ATG7 siRNA to inhibit autophagy in miRNA-122a mimic-transfected and miRNA-NC-transfected HK-2 cells treated with TGF-*β*1. Our results showed that both ATG7 siRNA and 3-MA treatments resulted in decreased expression of fibronectin, Col1a1, *α*-SMA, TGFBR1, LC3II/I, Beclin 1, and ATG7 and increased expression of E-cadherin, p62, and phosphor-mTOR, and AKT compared with the mRNA-NC or miRNA-122a mimic group (Figures 4(a)–4(c)). Moreover, the changes in fibronectin, Col1a1, *α*-SMA, TGFBR1, LC3II/I, E-cadherin, p62, p-mTOR, and p-AKT expression by 3-MA and ATG7 siRNA were more evident in miRNA-122a mimic-transfected HK-2 cells (Figures 4(a)–4(c)). In addition, the autophagic flux in miRNA-122a mimic-transfected and miRNA-NC-transfected HK-2 cells attenuated by 3-MA and ATG7 siRNA was monitored by a tandem fluorescence mRFP- (mCherry red fluorescent protein-) GFP-LC3 reporter system. The results indicated that the effect of miRNA-122a mimic on autophagic flux was significant (Figure 4(d)). These results indicate that miRNA-122a relieves renal fibrosis through the autophagy signaling pathway and autophagic flux.

### 3.5. miRNA-122a Mimic-Transfected MSC Treatment Improves Injured Kidney Architecture and Reduces Renal Fibrosis in UUO-Injured Mice

**Figure 5:**
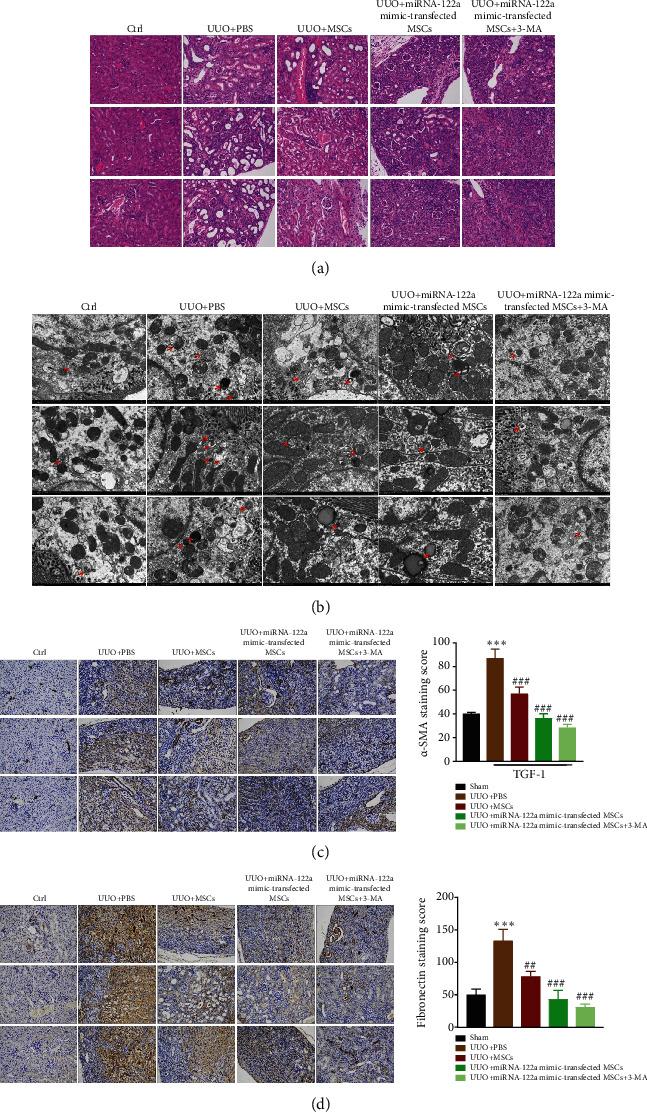
miRNA-122a mimic-transfected MSC treatment improves injured kidney architecture and reduces renal fibrosis in unilateral ureteral obstruction- (UUO-) injured mice. (a) Representative hematoxylin and eosin-stained sections of the sham group and UUO kidneys at 7 days postinjury treated with either PBS vehicle, miRNA-122a mimic-transfected MSCs, or miRNA-122a mimic-transfected MSCs+3-MA. (b) Autophagosomes were detected by TEM for each group of mice 24 h after within the border zone. Arrowhead, autophagosomes. (c, d) Representative photomicrographs of immunohistochemical staining of *α*-SMA (c) and fibronectin (d) in sham and UUO kidneys at 7 days postinjury treated with either PBS vehicle, miRNA-122a mimic-transfected MSCs, or miRNA-122a mimic-transfected MSCs+3-MA (magnification ×400).

Compared with the control group, UUO-induced hydronephrosis was characterized by decreased renal parenchyma, showing renal tubule expansion and interstitial expansion (Figure 5(a)). After treating with MSCs or miRNA-122a mimic-transfected MSCs, the UUO-injured kidneys exhibited reduced expansion of renal tubule, interstitial, and preservation of kidney architecture, which was more evident in UUO kidneys receiving miRNA-122a mimic-transfected MSCs (Figure 5(a)). However, the protective effect of miRNA-122a mimic-transfected MSCs on UUO-injured kidneys was enhanced by 3-MA treatment (Figure 5(a)). Compared with the control group, UUO stimulated autophagosome production in kidney tissue (Figure 5(b)). After treating with MSCs or miRNA-122a mimic-transfected MSCs, the UUO-induced autophagosome in kidney tissue was suppressed, which was further enhanced by 3-MA treatment (Figure 5(b)). These results indicate that miRNA-122a mimic-transfected MSC treatment improved injured kidney architecture in UUO-injured mice by inhibiting the autophagic process.

To observe the effect of miRNA-122a mimic-transfected MSC treatment on renal fibrosis, immunohistochemical staining of *α*-SMA and fibronectin in kidney tissue was performed. As a result, both MSC and miRNA-122a mimic-transfected MSC treatments resulted in a significant decrease in *α*-SMA and fibronectin protein localization in UUO-injured kidney tissue compared with UUO kidneys receiving vehicle alone (Figures 5(c) and 5(d)). Furthermore, 3-MA treatment further reduced *α*-SMA and fibronectin protein localization in UUO-injured kidney tissue (Figures 5(c) and 5(d)). These results indicate that miRNA-122a mimic-transfected MSC treatment reduces renal fibrosis in UUO-injured mice.

### 3.6. UUO-Induced Changes in the Levels of TGF-*β*1 and the Expression of Autophagy-Related Proteins, p-mTOR, and p-AKT Are Improved by miRNA-122a Mimic-Transfected MSC Treatment in UUO-Injured Mice

**Figure 6:**
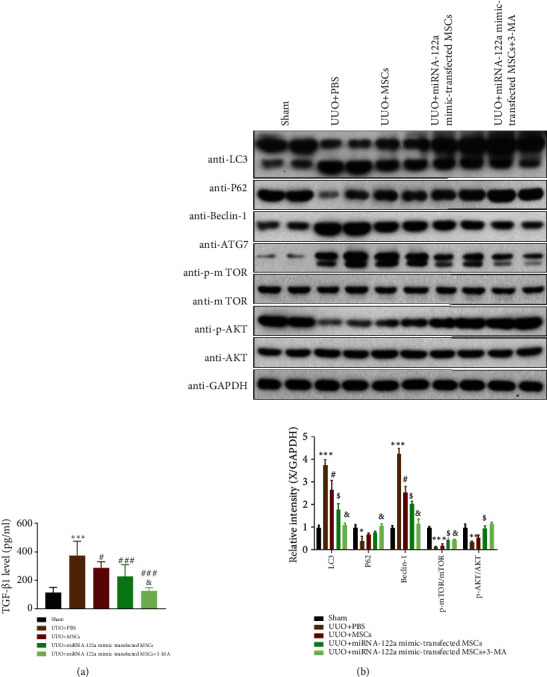
Unilateral ureteral obstruction- (UUO-) induced changes in the levels of TGF-*β*1, and expression of autophagy-related proteins, p-mTOR, and p-AKT was improved by miRNA-122a mimic-transfected MSC treatment in UUO-injured mice. (a) miRNA-122a mimic-transfected MSC treatment dramatically reduced the serum levels of TGF-*β*1 in the sham and UUO groups at 7 days postinjury treated with either PBS vehicle, miRNA-122a mimic-transfected MSCs, and miRNA-122a mimic-transfected MSCs+3-MA (*n* = 6/group). (b) LC3II/I, p62, Beclin 1, ATG7, mTOR, p-mTOR, AKT, and p-AKT protein expression was evaluated by western blotting in UUO kidney tissue, and the quantitative analysis is shown on the right (*n* = 3 independent experiments). The results are expressed as the mean ± SEM. Compared with the sham group, ^∗∗∗^*P* < 0.001, ^∗∗^*P* < 0.01, and ^∗^*P* < 0.05. Compared with the UUO+PBS group, ^###^*P* < 0.001, ^##^*P* < 0.01, and ^#^*P* < 0.05.

TGF-*β*1 is a key mediator in the development of kidney fibrosis. We measured the serum levels of TGF-*β*1. As shown in Figures 5(a) and 5(b), UUO-injured mice exhibited a marked increase in the level of TGF-*β*1 compared with those in the sham group. After receiving MSCs, miRNA-122a mimic-transfected MSCs, or miRNA-122a mimic-transfected MSC+3-MA treatment, the levels of TGF-*β*1 in UUO-injured mice were significantly decreased, which was more evident in the miRNA-122a mimic-transfected MSC+3-MA-treated group (Figure 6(a)). The levels of TGF-*β*1 in the miRNA-122a mimic-transfected MSC+3-MA-treated group were comparable with those in the sham group (Figure 6(a)). These results suggest that miRNA-122a mimic- transfected MSC treatment reduced kidney fibrosis and improved kidney function in UUO-injured mice.

To validate the expression of autophagy-related proteins, p-mTOR, and p-AKT affected by miRNA-122a mimic-transfected MSCs in vivo, western blot analysis was conducted to measure the relative expression of LC3II/I, p62, Beclin 1, p-mTOR, and p-AKT in the kidney tissue. As shown in Figure 6(b), MSCs, miRNA-122a mimic-transfected MSCs, or miRNA-122a mimic-transfected MSC+3-MA treatment in UUO mice all resulted in decreased expression of LC3II/I and Beclin 1 and increased expression of p62, p-mTOR, and p-AKT. These effects were most obvious in UUO mice receiving miRNA-122a mimic-transfected MSC+3-MA treatment. The results suggest that miRNA-122a mimic-transfected MSC treatment relieves kidney fibrosis of UUO-injured mice by regulating mTOR signaling and downstream autophagy-related protein expression.

## 4. Discussion

Renal interstitial fibrosis is the pathological basis and final pathway of renal disease. Under the action of multiple factors, it causes inflammatory infiltration, fibroblast proliferation, and deposition of extracellular depositional matrices in the renal interstitium [20], which is one of the characteristics of renal failure. miRNAs play an important role in kidney development and maintenance of kidney function. They are the main regulator of cell biological functions during the occurrence and development of fibrosis [21, 22]. Reports have confirmed that miRNAs are closely related to renal interstitial fibrosis, suggesting that miRNA-targeted therapy can inhibit or block fibrosis to avoid renal replacement therapy [23, 24]. MSC can transfer endogenous miRNAs through exosomes. The therapeutic strategy of coupling MSC and miRNA has been regarded as a promising and effective antifibrosis treatment for kidney injury [25]. miRNA-122 is related to fibrogenesis [19], suggesting that miRNA-122 may be implicated in the renal fibrosis. In the present study, we showed that a strategy for relieving fibrosis of kidney tubular epithelial cells via MSC-derived exosome transfer of therapeutic miRNA-122 regulated the autophagy and mTOR-related signaling pathways.

Among the many factors that promote fibrosis, TGF*β* is considered to be a key cytokine involved in tubular sclerosis and tubular epithelial fibrosis [26]. TGF*β* overexpression can promote the accumulation of extracellular matrix proteins in the mesangium, can reduce its degradation, and can directly stimulate the transcription of downstream effector genes. This regulates the growth and secretion of fibroblasts and mesangial extracellular matrix and promotes glomerular sclerosis and renal interstitial fibrosis [27]. Currently, many studies have demonstrated the efficacy of MSCs as a valuable tool for improving renal interstitial fibrosis [11–13]. Moreover, the application of MSC for the treatment of kidney disease has no adverse side effects in humans. This is consistent with our in vitro results showing that MSC supernatant treatment reduces the expression of *α*-SMA, Col1a1, and fibronectin and increases E-cadherin expression in TGF-*β*1-treated HK-2 cells. This was further confirmed in a UUO in vivo model, which resulted in the improvement of renal interstitial fibrosis by MSCs. The key point of MSC treatment is to target the paracrine mechanism, rather than the MSC itself. One study showed that exosomes derived from MSCs reversed the deterioration of renal function in the UUO model, and its therapeutic effect was superior than that of MSC's alone [14]. Our study also demonstrated that MSC-derived exosomes normalize related fibrotic genes (*α*-SMA, Col1a1, fibronectin, and E-cadherin) better than MSCs, and these improvements can be reversed by treatment with GW4869, an exosome inhibitor, proving that MSC exerts its anti-kidney fibrosis effect through exosomes.

Many miRNAs are closely associated with the TGF-*β* signaling pathway and participate in the formation and regulation of extracellular matrix, EMT, and fibroblast-myofibroblast activation [28]. miRNA-122 has been found to increase the levels of the fibrotic factors, collagen 1*α*1, collagen 1*α*2, and TGF-*β*1 in the NAFLD cell model [29]. Administration of a miRNA-122 inhibitor reduced the key TGF-*β*-induced fibrotic signaling pathway, promoted collagen synthesis, and stimulated fibrinogenesis, consequently resulting in the accumulation of fibroblasts and extracellular matrix in hypertension and cardiovascular diseases [30, 31]. In this study, sRNA next-generation sequencing identified miRNA-122 as the most upregulated miRNA affected by MSC-exo in TGF-*β*1-treated HK-2 cells. Furthermore, the results from TGF-*β*1-treated HK-2 cells and the UUO model both demonstrated that miRNA-122a mimic treatment significantly reduces the expression of *α*-SMA, Col1a1, and fibronectin genes and enhances E-cadherin expression. These results suggest that miRNA-122a play a role in renal fibrosis amelioration.

Autophagy degrades excess proteins or damaged organelles to power cells and promote cell survival. MSC can regulate host cell autophagy through the mTOR signaling pathway. The effect of autophagy in renal interstitial fibrosis is not exactly the same. Kim et al. [32] found that 3-MA pretreatment further enhances the degree of renal interstitial fibrosis in UUO model rats, suggesting that autophagy is involved in renal interstitial fibers and exerts a protective effect in renal interstitial fibrosis. Another study from Bernard et al. [33] found that starvation induced increased autophagy activity in fibroblasts, which was accompanied by increased expression of *α*-SMA and type I and type III collagen and increased cell differentiation. Application of 3-MA or siRNA ATG7 to inhibit autophagy weakens the degree of fibroblast differentiation, suggesting that autophagy can promote fibroblast differentiation and thereby aggravate the degree of organ fibrosis. However, Livingston et al. found that ATG7 knockout inhibited UUO-induced autophagy activation and further reduced the expression of renal interstitial fibrosis-related indicators, such as FN, *α*-SMA, and extracellular matrix collagen, resulting from UUO. This indicates that inhibiting autophagy significantly improves renal interstitial fibrosis and autophagy plays a damaging role in renal interstitial fibrosis [34]. In our study, ATG7 siRNA and 3-MA treatment resulted in decreased expression of fibronectin, Col1a1, *α*-SMA, TGFBR1, LC3II/I, Beclin 1, and ATG7 and increased expression of E-cadherin, p62, and p-mTOR and AKT in TGF-*β*1-treated HK-2 cells transfected with miRNA-122a mimic. These results suggest that the inhibition of autophagy can further strengthen the pathway of miRNA-122a to mTOR signaling and cause the inhibition of autophagy in renal tubular duct epithelial cells, which is consistent with the findings of Song et al. [35]. Our results also suggest that autophagy has a damaging effect in renal interstitial fibrosis. Nonetheless, there are still some aspects that may need further studies. Firstly, 3-MA is not a specific autophagy inhibitor; high doses of 3-MA may activate other kinases and signaling pathways, thereby exerting other biological effects besides autophagy inhibition. Therefore, future studies are needed to explore whether different doses of 3-MA have different effects on autophagy inhibition by miRNA-122a. Secondly, a large number of studies have confirmed that ATG7 has an important role independent of other ATGs in a variety of diseases. Thus, the interference of other ATG genes on autophagy inhibition by miRNA-122a also warrants further study. Thirdly, while our results support the role of miRNA-122a in protecting against interstitial fibrosis, to confirm if this is the case, using MSCs transfected with miRNA-122a inhibitor to see whether inhibition of miRNA-122a of MSCs abrogates its protective effects in the UUO mice remains to be further investigated. Fourthly, tubular epithelial cells would be ideal to study the MSCs in fibroblast cells. Nonetheless, it has been reported that in addition to tubular epithelial cells, glomerular mesangial cells and kidney interstitial fibroblasts cells are also involved in the pathogenesis of kidney fibrosis [36, 37]. It would be necessary to explore the effect of MSCs and miRNA-122a-transfected MSCs in these cell line models in the future.

## 5. Conclusion

In conclusion, our results demonstrate that MSC treatment improves injured kidney architecture and reduces renal fibrosis by autophagic inhibition and by interfering with the mTOR signaling pathway via excreted exosomes containing mainly miRNA-122a. Collectively, these findings provide new insights into the mechanisms through which miRNAs secreted by MSC-derived exosomes affect renal fibrosis which will lead to strategies for the treatment of renal fibrosis.

## Figures and Tables

**Table 1:** The top ten upregulated miRNAs in the mesenchymal stem cell- (MSC-) exo vs. the MSC supernatant+GW4869 group.

miRNA ID	*P* value	Log2FC	Regulated
hsa-miR-122-5p	0.0000014722	5.052374951	Up
hsa-miR-3196	0.0000000001	3.985461731	Up
hsa-miR-663a	0.0008696417	3.044774648	Up
hsa-miR-320e	0.0309679928	2.685565385	Up
hsa-miR-487b-3p	0.0390985517	2.663385439	Up
hsa-miR-3195	0.0000000607	2.627461493	Up
hsa-miR-6836-3p	0.0389217735	2.249343498	Up
hsa-miR-6780a-5p	0.0386224149	2.238607839	Up
hsa-miR-411-3p	0.0415914722	2.019926675	Up
hsa-miR-4454	0.0000027959	1.980996124	Up

## Data Availability

The datasets used and/or analysed during the current study are available from the corresponding author on reasonable request.

## Supplementary Materials

Supplementary 1 Figure S1: characterization of mesenchymal stem cells (MSC). (A) Flow cytometry verified MSC surface antigens. (B) The extraction of MSC exosomes verified by electron microscopy (B), NTA (C), and western blot analysis (D). PKH26 staining to observe HK-2 cell exosomal uptake (E).

Supplementary 2 Table S1: primers for the target genes.

Supplementary 3 Table S2: the antibody used in this study.

Supplementary 4 Table S3: list of all miRNA identified from six sRNA libraries.

Supplementary 5 Table S4: miRNAs expressed in TGF-*β*1-treated HK-2 cells treated with mesenchymal stem cell (MSC) supernatant+GW4869 (GW4869-1, GW4869-2, and GW4869-3) and treated with MSC-exo (exo-1, exo-2, and exo-3).

Supplementary 6 Table S5: differentially expressed miRNAs between the mesenchymal stem cell (MSC)+GW4869 and MSC-exo groups.
