# Supplementary figures and images for: Mesenchymal Stem Cells Alleviate Renal Fibrosis and Inhibit Autophagy via Exosome Transfer of miRNA-122a
Source: Stem Cells Int. 2022 Jul 7;2022:1981798. doi: 10.1155/2022/1981798 (PMC9289760; doi:10.1155/2022/1981798)

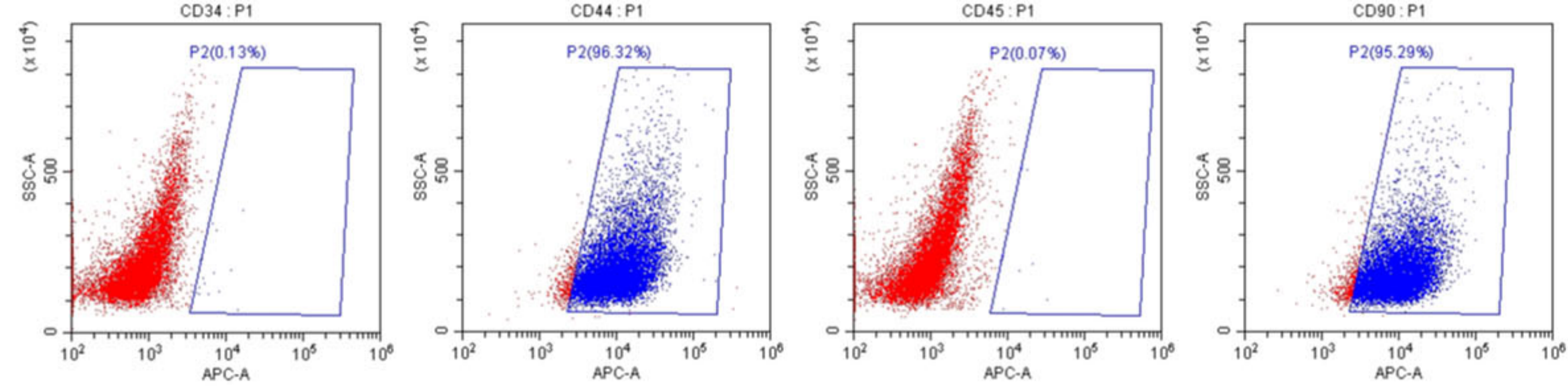

**A**

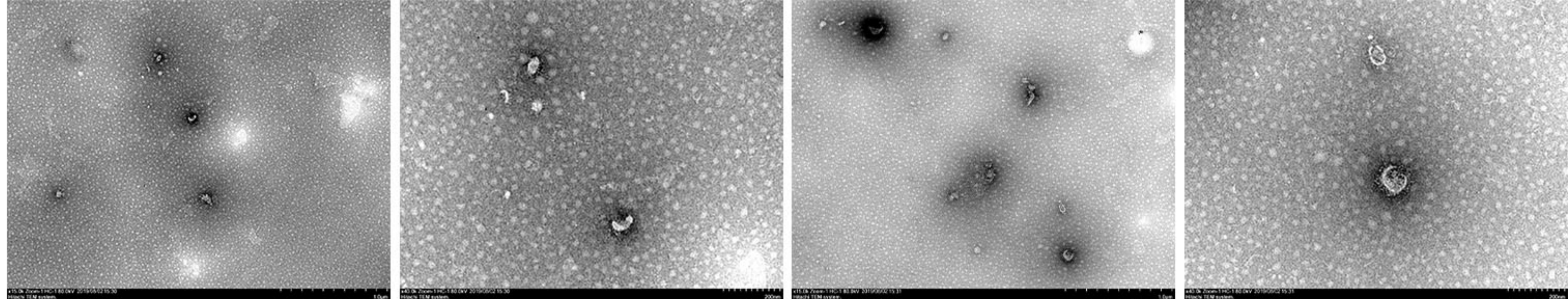

**B**

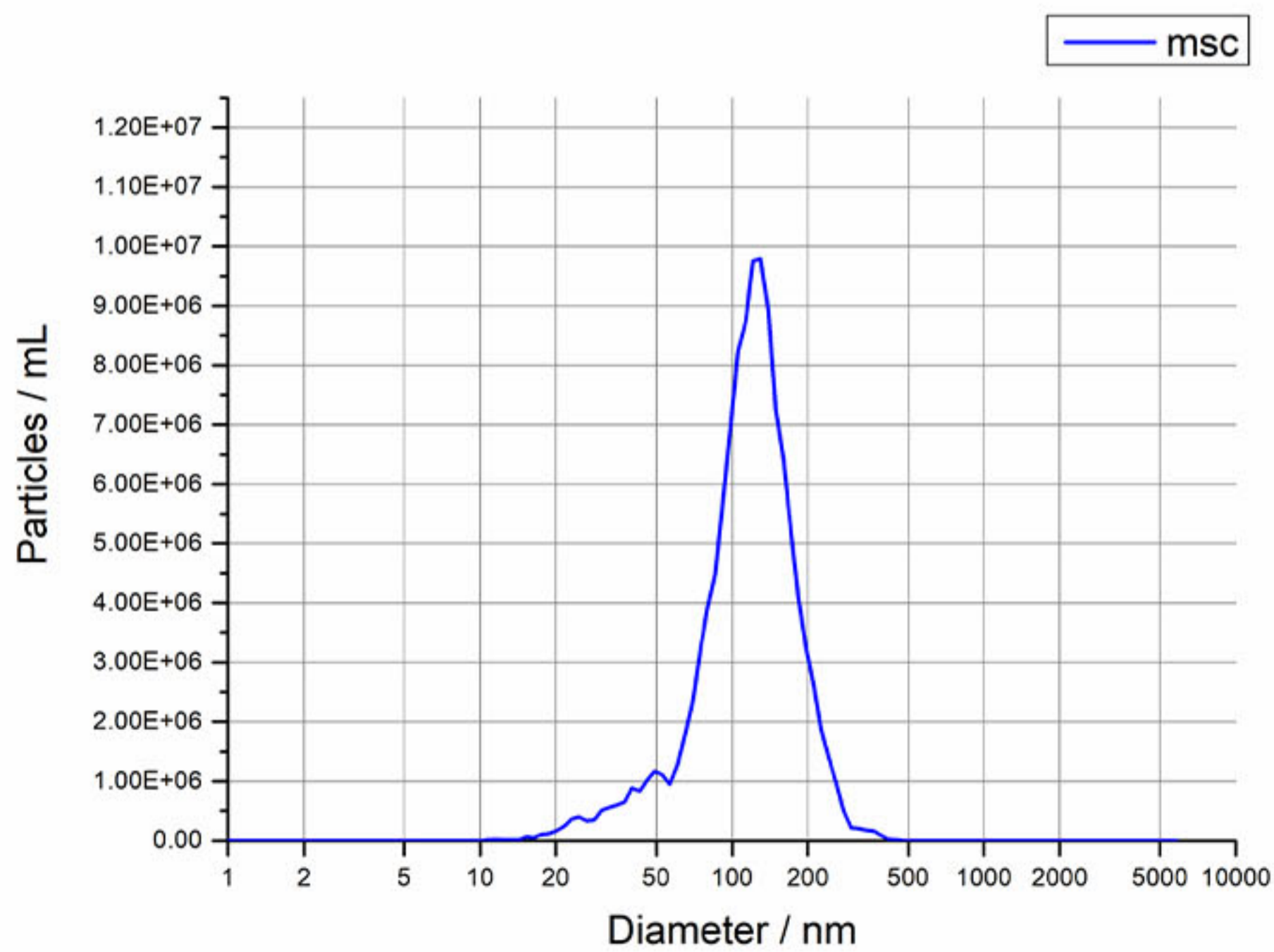

**C**

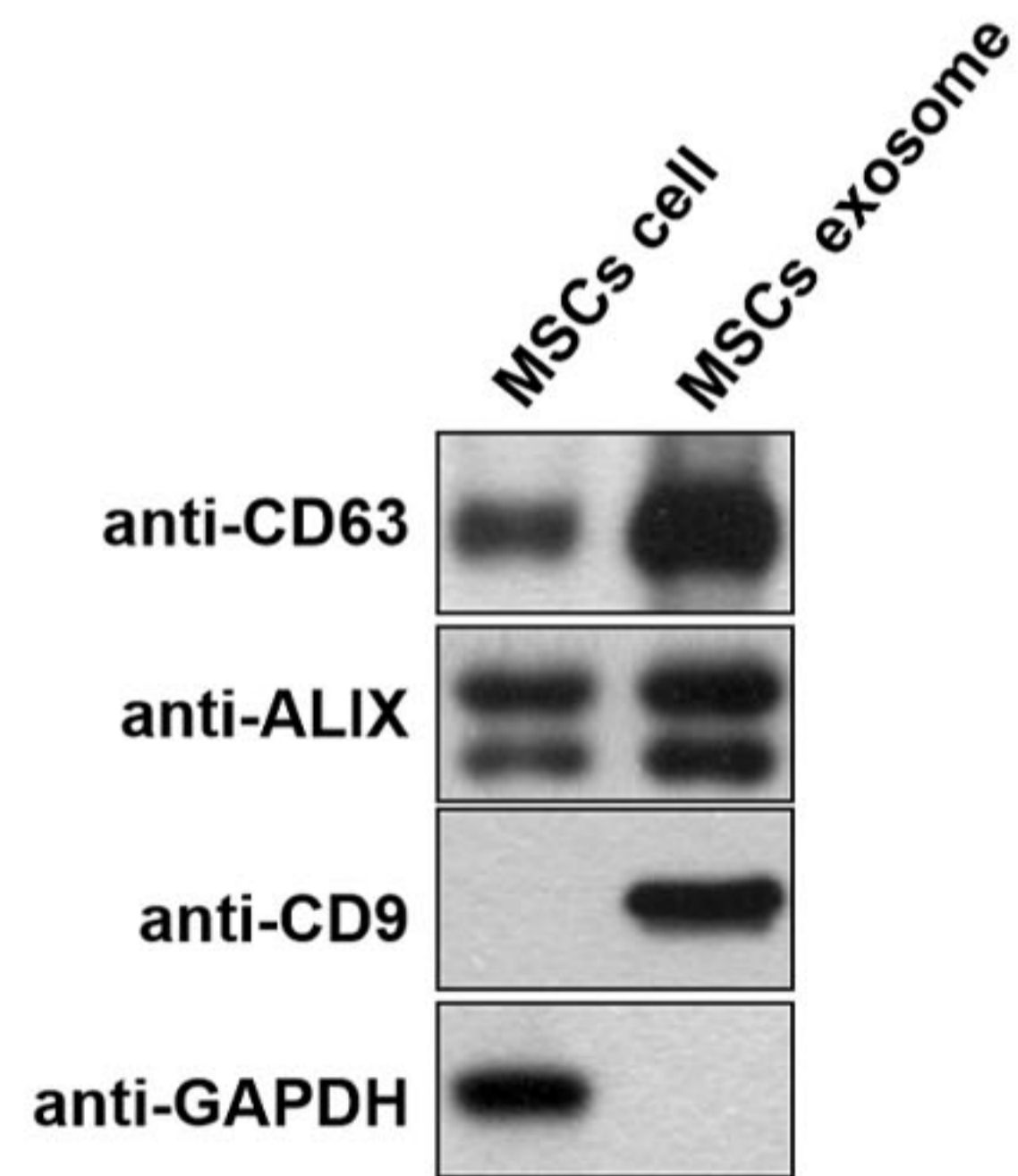

**D**

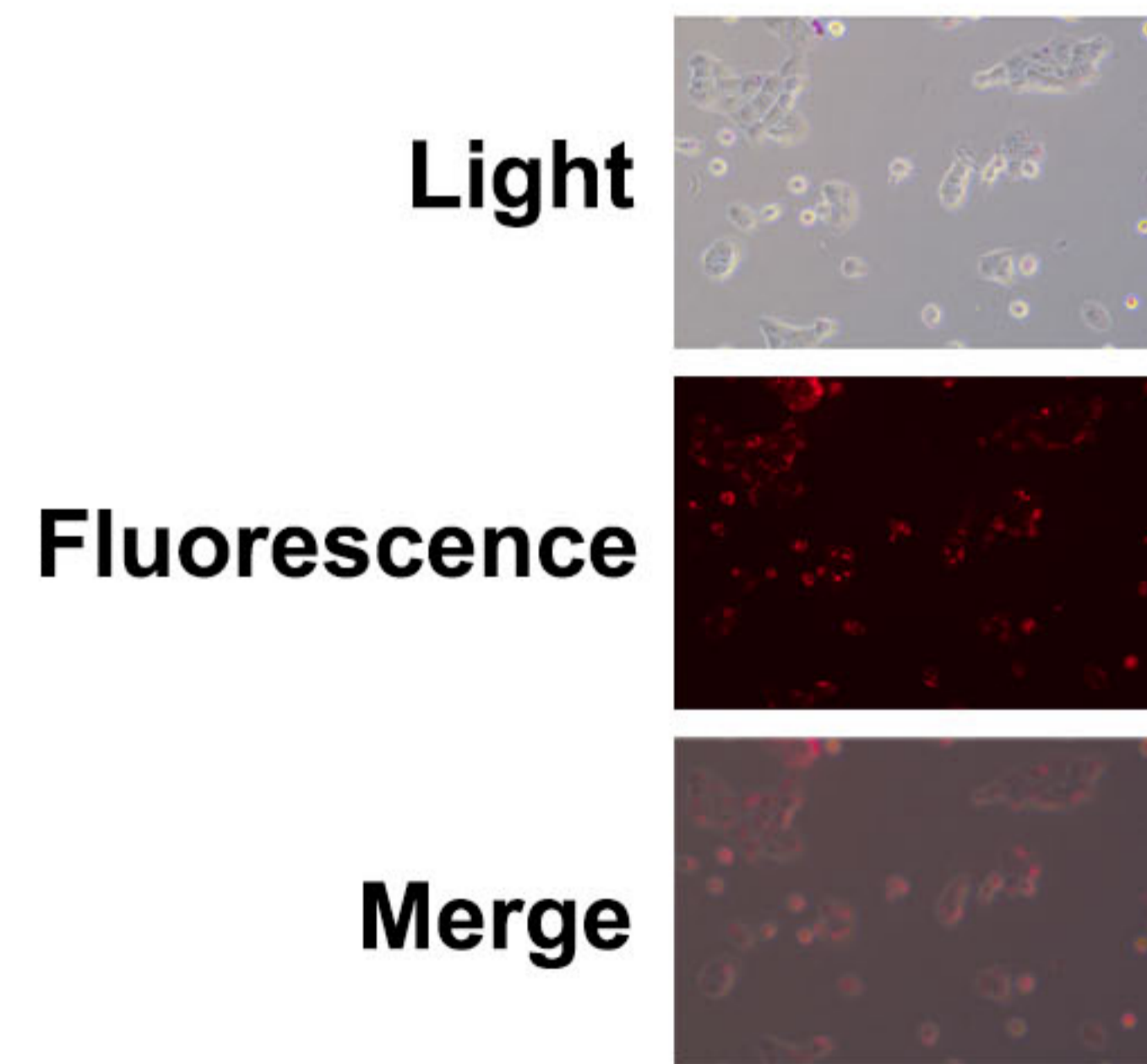

**E**

Supplement: Supplementary 1 — Figure S1: characterization of mesenchymal stem cells (MSC). (A) Flow cytometry verified MSC surface antigens. (B) The extraction of MSC exosomes verified by electron microscopy (B), NTA (C), and western blot analysis (D). PKH26 staining to observe HK-2 cell exosomal uptake (E). [file 1981798.f1.pdf]
