# Supplementary material for: Mesenchymal Stem Cells Alleviate Renal Fibrosis and Inhibit Autophagy via Exosome Transfer of miRNA-122a
Source: Stem Cells Int. 2022 Jul 7;2022:1981798. doi: 10.1155/2022/1981798 (PMC9289760; doi:10.1155/2022/1981798)
Supplement: Supplementary 2 — Table S1: primers for the target genes. [file 1981798.f2.doc]

Table S1 The primers for the target genes

| Primer | Sequence (5’→3’) |
| --- | --- |
| H-TGFBR1-F | CACAGAGTGGGAACAAAAAGGT |
| H-TGFBR1-R | CCAATGGAACATCGTCGAGCA |
| H-a-SMA-F | CTGCTGAGCGTGAGATTGTC |
| H-a-SMA-R | TCAAGGGAGGATGAGGATGC |
| H-Col1a1-F | GTGACCTCAAGATGTGCCAC |
| H-Col1a1-R | CTTGTCCTTGGGGTTCTTGC |
| H-Fibronectin-F | AGGAAGCCGAGGTTTTAACTG |
| H-Fibronectin-R | AGGACGCTCATAAGTGTCACC |
| H-E-cadherin-F | CGAGAGCTACACGTTCACGG |
| H-E-cadherin-R | GGGTGTCGAGGGAAAAATAGG |
| h-GAPDH-F | GGAGCGAGATCCCTCCAAAAT |
| h-GAPDH-R | GGCTGTTGTCATACTTCTCATGG |
