# Supplementary material for: Mesenchymal Stem Cells Alleviate Renal Fibrosis and Inhibit Autophagy via Exosome Transfer of miRNA-122a
Source: Stem Cells Int. 2022 Jul 7;2022:1981798. doi: 10.1155/2022/1981798 (PMC9289760; doi:10.1155/2022/1981798)
Supplement: Supplementary 3 — Table S2: the antibody used in this study. [file 1981798.f3.docx]

Table S2. The antibody used in this study

| **Antibody** | **Dilution** | **Company** | **Cat. No** |
| --- | --- | --- | --- |
| TGFBR1 | 1:1000 | abcam | ab235178 |
| α-SMA | 1:1000 | abcam | ab5694 |
| Col1a1 | 1:1000 | abcam | ab34710 |
| Col1a4 | 1:1000 | abcam | ab6586 |
| Fibronectin | 1:1000 | abcam | ab23750 |
| E-cadherin | 1:1000 | abcam | ab76055 |
| LC3 | 1/2000 | abcam | ab192890 |
| p62 | 1/10000 | abcam | ab109012 |
| Beclin 1 | 1/2000 | abcam | ab207612 |
| ATG7 | 1/10000 | abcam | ab133528 |
| pmTOR | 1/10000 | abcam | ab109268 |
| mTOR | 1/10000 | abcam | ab134903 |
| pAKT | 1/5000 | abcam | ab81283 |
| AKT | 1/10000 | abcam | ab179463 |
| GAPDH | 1:20000 | cst | #5174 |
| Anti-mouse IgG (HRP) | 1:5000 | cst | #7076 |
| Anti-rabbit IgG (HRP) | 1:5000 | cst | #7074 |
| CD34 |  | abcam | ab81289 |
| CD44 |  | abcam | ab189524 |
| CD45 |  | abcam | ab40763 |
| CD90 |  | abcam | ab92726 |
| CD63 |  |  |  |
| Alix |  |  |  |
| CD9 |  |  |  |
